# Gleanings in Science and Medicine

**Published:** 1893-07-01

**Authors:** 


### Gleanings in Science and Medicine.

A new antiseptic is announced. In the course of some experiments at the Thorncliffe Collieries, near Sheffield, Mr. J. H. Worrall found that one of the products of the Thorncliffe patent coke ovens was a hitherto unknown oil, which yields an antiseptic that is said to be more powerful than pure carbolic acid. It is insoluble in water for all practical purposes, and has a boiling point higher than that of carbolic acid. In addition to its disinfectant qualities, it is said to be a germicide of remarkable power, apparently rivalling salicylic acid in that respect. Izal is the name which has been given to this new antiseptic, which, by the bye, is said to be non-poisonous.

---

The *Scientific American* describes the engine which was used in the first regular trip of a locomotive and train in the State of New York. It weighed just six tons. The driving wheels, four in number, were four feet six inches in diameter, and the cylinders, two in number, were five and a half inches in diameter by sixteen inches stroke. This primitive engine drew a train of three cars, shaped like the old-fashioned stage coaches. The trial trip took place on August 9th, 1831. A tin horn was blown as the signal for starting. The fuel used was dry pitch pine. The passengers were half smothered by smoke and covered with sparks from the chimney. "They raised their umbrellas to protect themselves, but the covers were soon burnt off, and the passengers busied themselves in putting out, in each others clothes, the fire started by the hot cinders." The couplings were evidently of a very simple kind, for when the train stopped there was a disagreeable jerk, which was corrected by taking rails from a fence, wedging them between the cars, and tying them fast. An exact reproduction of this car is among the exhibits at the great show at Chicago; and beside it stands a huge sixty-two ton locomotive, lately built for the New York Central and Hudson River Railway, with all the modern improvements. The two exhibits must be an interesting object lesson in the marvellous progress which has been made in engineering science during the last half century.

